# IgE Anti‐Beta Coronaviruses Serology in Napoleon Soldiers, France

**DOI:** 10.1002/jmv.70800

**Published:** 2026-01-13

**Authors:** Nor El Houda Merrouche, Gérard Aboudharam, Sandrine Thiol, Elodie Terrer, Jacques Fantini, Michel Drancourt, Hamadou Oumarou Hama

**Affiliations:** ^1^ IHU‐Méditerranée Infection Marseille France; ^2^ MEPHI Aix‐Marseille Université Marseille France; ^3^ Pôle Odontologie Assistance Publique – Hôpitaux de Marseille Marseille France; ^4^ École de Médecine Dentaire Aix‐Marseille‐Université Marseille France; ^5^ INRAP/Centre Archéologique de Reims Université de Reims Champagne‐ Ardenne Reims France; ^6^ INSERM UA 16 Aix Marseille Université Marseille France

**Keywords:** coronavirus, dental pulp, IgE, nucleocapsid, paleoserology

## Abstract

To compare the repertoire of anti‐beta‐Coronavirus antibodies detected in dental pulp samples (systemic immunity) collected from individuals from the early 19th century previously investigated for dental calculus (local immunity) serological response, We investigated 10 dental pulp samples collected from 10 individuals excavated from a 1810–1813 military site in Charleville‐Mézières, France. The samples had previously been investigated for dental calculus serology. Dental pulp serology performed under a mini‐blot format, incorporated one positive and one negative control, and conjugated antibodies against the five classes of immunoglobulins. Dental pulp IgE serological response reliability was assessed by *in silico* analyses. Controls yielded expected results. Anti‐Coronavirus antibodies were detected in three individuals, comprising anti‐beta Coronavirus IgE in three individuals, IgG in two individuals, and IgA in one individual. IgA and IgG anti‐alpha Coronavirus were each detected in one individual. These results agreed with those previously obtained from the same 10 individuals with anti‐beta‐Coronavirus pooled IgG/IgA/IgM dental calculus paleoserology. Dental pulp paleoserology confirmed Coronavirus exposure in three individuals from the start of the 19th century in France. Translating these data into the modern medical literature, we propose that two centuries ago, some individuals suffered a yet unidentified beta‐Coronavirus infection.

## 1 Introduction

In 1810–1813, the town of Charleville‐Mézières, located in the northeast of France, was a garrison for the army of French Emperor Napoleon the First [1]. Ten soldiers exhumed from that archaeological site had previously been investigated by paleoserology for anti‐Coronavirus antibodies, following the extraction of paleosera from dental calculus samples. This investigation yielded the detection of pooled IgA/IgM/IgG antibodies against the beta‐Coronavirus SARS‐CoV‐2 antigen in one individual and against the beta‐Coronavirus HCoV OC43 antigen in a second individual. Automated Western blot assays confirmed SARS‐CoV‐2 nucleocapsid protein antibodies [2]. As dental calculus embeds an average 10‐day pre‐mortem memory of the oral and nasopharyngeal cavity biology, this data indicate that these individuals certainly developed local oral cavity immunity against an as‐yet unknown beta‐Coronavirus, testifying to these individuals' exposure to such a beta‐Coronavirus. This data however left unknown whether these individuals developed systemic immunity against this beta‐Coronavirus in addition to local immunity.

In order to answer this question, we further investigated systemic paleoserological responses in these individuals by testing the paleoserum samples extracted from the dental pulp tissues, an organ recognised as containing blood drops from the time of death and thus indicating systemic immunity, if any [3].

## 2 Materials and Methods

### 2.1 Dental Pulp Specimens

Dental pulp samples were collected from the 10 individuals exhumed from the 1810–1813 Charleville‐Mézières site in agreement with the laws and regulations in France at the time of the study, as previously reported [2].

### 2.2 Paleoserology

**FIGURE 1:**
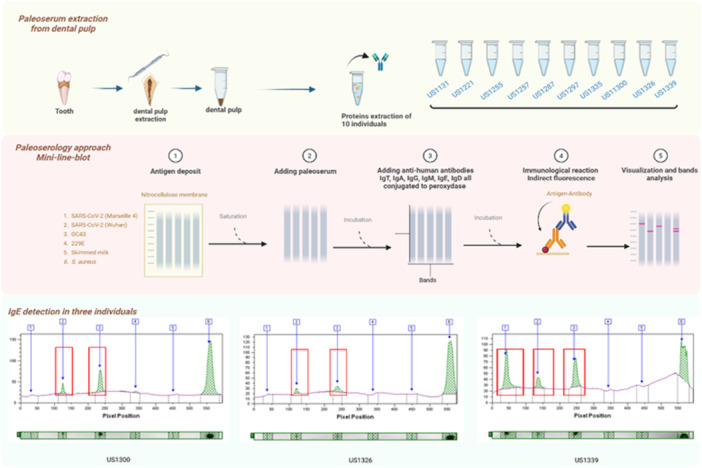
The figure illustrates the key steps from sample preparation to detection of immunological reactions; it shows the reactivity of immunoglobulin E (IgE) from three individuals (US1300, US1326, and US1339) to the SARS‐CoV‐2 coronavirus antigens (Marseille 4 and Wuhan) and OC43 antigen. The graphs at the bottom of the figure show the quantification of these reactions, with the intensity peaks (red boxes) corresponding to the presence of specific IgE reacting to the viral antigens tested.

Paleoserum samples were extracted from ancient dental pulp tissues as previously reported [4]. More specifically, paleoserology incorporated heat‐inactivated (65°C for 1 h) SARS‐CoV‐2 MI2 (Wuhan genotype), SARS‐CoV‐2 2096 (Marseille 4 genotype), OC43 and 229 E Coronavirus, and peroxidise‐conjugated immunoglobulins against human pooled IgA/IgM/IgG (Jackson Immuno Research, Ely, United Kingdom), IgA, IgG, IgM, IgE, and IgD (SouthernBiothech, Birmingham, USA). Serology reactions incorporated *Staphylococcus aureus* (*S. aureus*) as a positive control, non‐specifically catching any immunoglobulins, and skimmed milk as a negative control [5] (Figure 1).

### 2.3 Structural Analyses

All simulations were performed with the Hyperchem suite and visualised with Molegro Molecular Viewer, as described previously [6]. In order to interpret the results obtained, we developed an original approach based on the detection of IgE epitopes in the NC protein of Coronaviruses. For this, we developed our own algorithm, taking into account the following criteria: (i) localisation of the epitope on the surface of the protein, which suggests an over‐representation of polar amino acids in the motif area; (ii) the presence of a turn in the motif, induced by one or more Gly and Pro residues; and (iii) a negative surface electrostatic potential, requiring the presence of Asp and/or Glu residues. Our algorithm is based on previously published biochemical characterisation of IgE epitopes and careful analysis of available databases [7, 8]; Khatri et al. 2022 [9].

## 3 Results

Both controls yielded expected results. The negative control (skimmed milk) was negative for all antigens and immunoglobulin classes tested and confirmed the absence of non‐specific reactivity, while the positive control (*S. aureus*) showed reactivity across all antibody classes, confirming the reliability of the tests. Pooled IgA/IgM/IgG detected in three individuals corresponded to IgG and IgA (in two individuals). Interestingly, the three individuals yielded IgE against two or three of the beta‐Coronavirus, but not against alpha‐Coronavirus 229E (Figure 1), while IgD were never detected (Table 1). In detail, paleoserum US1300 showed the presence of IgE antibodies reactive against two beta‐Coronaviruses: SARS‐CoV‐2 (Wuhan) and OC43. No reactivity was detected with the other antigens tested (Supporting Information Table 1). Paleoserum US1326 showed reactivity against the three beta‐Coronaviruses, SARS‐CoV‐2 (Marseille 4), SARS‐CoV‐2 (Wuhan), and OC43. Pooled IgA/IgM/IgG antibodies were detected against the SARS‐CoV‐2 (Marseille 4) antigen, while pooled IgA/IgM/IgG, IgG, and IgE antibodies were detected against the SARS‐CoV‐2 (Wuhan) antigen. Only IgE was detected against the OC43 antigen, and IgG were detected against the 229E antigens (Supporting Information Table 2). Paleoserum US1339 showed broad reactivity against all antigens tested. IgG and IgE antibodies were detected in response to the SARS‐CoV‐2 (Marseille 4) antigen, and pooled IgA/IgM/IgG and IgE were detected against both the beta‐Coronavirus SARS‐CoV‐2 (Wuhan) antigen and OC43 antigen, while reactivity was observed with IgA antibodies against the 229E antigen (Supporting Information Table 3). Paleosera from the other individuals (US1131, US1221, US1255, US1257, US1287, US1297, and US1335) were found to be completely negative for all tested Coronavirus antigens, with no antibodies detected for any of the immunoglobulin classes (Supporting Information Tables 4–10).

**TABLE 1:** Spot‐blot assay results for three paleosera extracted from three dental pulp samples collected from four individuals exhumed from the 19th century site of Charleville‐Mézières, France, reacting with anti‐human antibodies pooled IgA/IgM/IgG (herein referred as IgT), IgA, IgG, IgM, IgE, and IgD by comparison with *S. aureus* as a positive control and skimmed milk as a negative control.

Antigens	SARS‐CoV‐2 (Marseille 4)						SARS‐CoV‐2 (Wuhan)						OC43						229E					
Antibodies samples	IgT	IgA	IgG	IgM	IgE	IgD	IgT	IgA	IgG	IgM	IgE	IgD	IgT	IgA	IgG	IgM	IgE	IgD	IgT	IgA	IgG	IgM	IgE	IgD
Paleoserum US1300	—	—	—	—	—	—	—	—	—	—	+	—	—	—	—	—	+	—	—	—	—	—	—	—
Paleoserum US1326	+	—	—	—	—	—	+	—	+	—	+	—	—	—	—	—	+	—	—	—	+	—	—	—
Paleoserum US1339	—	—	+	—	+	—	+	—	—	—	+	—	+	—	—	—	+	—	—	+	—	—	—	—
Negative control (skimmed milk)	—	—	—	—	—	—	—	—	—	—	—	—	—	—	—	—	—	—	—	—	—	—	—	—
Positive control (*S. aureus*)	+	+	+	+	+	+	+	+	+	+	+	+	+	+	+	+	+	+	+	+	+	+	+	+

**FIGURE 2:**
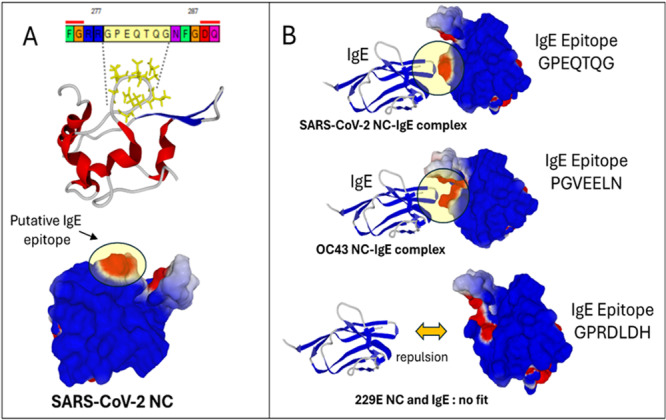
IgE epitope prediction in the three Coronaviruses SARS‐CoV‐2, OC43, and 229E. (A) Prediction of a major IgE epitope on the surface of the SARS‐CoV‐2 NC protein. The amino acid sequence of the epitope is indicated in the top panel. The loop structure of the epitope is visible in the middle panel. The bottom panel shows the electronegative surface potential of the epitope (red, electronegative; blue, electropositive; and white, neutral). A translucent yellow disc was superimposed to better visualise the surface potential of the IgE epitope. (B) The top panel shows a functional IgE‐NC complex for SARS‐CoV‐2. The middle panel shows a similar complex for the IgE epitope of OC43 inserted in the SARS‐CoV‐2 NC protein context. In both cases, a translucent yellow disc was superimposed to better visualise the surface potential of the IgE epitope and its accessibility for the antibody. The bottom panel shows the absence of fit between the IgE epitope of 229E NC protein inserted in the SARS‐CoV‐2 context, due to electrostatic repulsion.

Analysis of the SARS‐CoV‐2 NC protein sequence using a home‐designed algorithm made it possible to identify the GPEQTQG motif, meeting the required criteria, further localised in the 3D‐structure of the protein (Figure 2A). This motif does not exist in the OC43 and 229E Coronaviruses, which have their own IgE epitopes (Figure 2B). Since there is no available structure for the NC proteins of these two Coronaviruses, we created *in silico* chimeric proteins expressing these epitopes in the context of the 3D‐structure of the SARS‐CoV‐2 NC protein. We then reconstructed an antigen‐IgE complex for the SARS‐CoV‐2 NC protein. In this case, the complex appears clearly functional with excellent accessibility of the IgE epitope for the antibody (Figure 2B). Moreover, we found that the IgE epitope of OC43 is fully accessible to the antibody, while the 229E epitope is partially masked by an electropositive region, which might be responsible for an electrostatic repulsion and no fit between the protein and the antibody.

## 4 Discussion

While confirming the exposure of individuals exhumed from the early 19th century site of Charleville‐Mézières to beta‐Coronavirus, which we previously reported based on dental calculus serology testifying to a local, pharyngeal exposure to the virus [2], the present data further indicate a systemic serological response indicative of an infection in these individuals. We detected an IgE response against beta‐Coronavirus in the dental pulp, whereas IgE was not investigated in the dental calculus in our previous investigation [2]. An IgE systemic response against SFL3 and NFL antigens has been reported in 90% to 100% of patients infected with SARS‐CoV‐2 [10]. Accordingly, the SARS‐CoV‐2 nucleocapsid was reported to induce specific IgE, while no specific epitope had been reported [11]. Here, structural analysis prompted by our discovery of an IgE response in ancient individuals suggested that the IgE epitope characterised in the NC protein of the SARS‐CoV‐2 virus may originate from an ancestral epitope shared, at the structural level, by the ancestors of certain Coronaviruses. This would explain the cross‐reaction of antibodies detected in the dental pulp of Napoleonic soldiers on antigen‐like samples of SARS‐CoV‐2 and OC43. In contrast, the absence of a cross‐reaction with antigenic samples of the Coronavirus 229E suggests a lower accessibility of IgE epitopes by antibodies generated by old Coronavirus infections. The interpretation of IgE anti‐Coronavirus remains, however, controversial: while it was shown that an immune response characterised by the production of IgE could be a biomarker for the severity of COVID‐19, including death, as patients with severe COVID‐19 had significantly higher levels of IgE targeting the SARS‐CoV‐2 nucleocapsid [11], other studies did not find such a correlation [12, 13]. Therefore, the interpretation of data herein reported in two centuries‐old specimens cannot be assessed.

This study reinforces the interest of paleoserology studies coupling dental calculus and dental pulp to directly investigate the panel of five anti‐human immunoglobulins, with anti‐IgD being currently considered as a negative control, because no IgD response has ever been reported against a systemic pathogen.

## Author Contributions

**Nor El Houda Merrouche:** conceptualisation (equal), formal analysis (equal), funding acquisition (lead), investigation (lead), methodology (equal), visualisation (equal), writing – original draft (lead). **Gérard Aboudharam:** conceptualisation (equal), formal analysis (equal), funding acquisition (equal), funding acquisition (lead), validation (equal), supervision (equal), writing – review and editing (equal). **Sandrine Thiol:** investigation (equal), resources (equal), writing – review and editing (equal). **Elodie Terrer:** conceptualisation (equal), funding acquisition (equal), writing – review and editing (equal). **Jacques Fantini:** conceptualisation (equal), formal analysis (equal), methodology (equal), resources (equal), writing – review and editing (equal). **Michel Drancourt:** conceptualisation (lead), formal analysis (equal), investigation (equal), methodology (equal), validation (lead), visualisation (equal), supervision (equal), original draft (lead), writing – review and editing (equal). **Hamadou Oumarou Hama:** conceptualisation (equal), formal analysis (equal), investigation (equal), methodology (equal), validation (equal), visualisation (lead), supervision (equal), original draft (equal), writing – review and editing (equal).

## Conflicts of Interest

The authors declare no conflicts of interest.

## Supporting information


**Supplementary Table 1:** Spot‐blot test results for two paleosera extracted from a dental pulp sample and a dental calculus sample taken from one individual US1300 exhumed from the Charleville‐Mézières site, France, in the 19^th^ century (1810‐1813), reacting with anti‐ human antibodies compared with *Staphylococcus aureus* as a positive control and with skimmed milk as a negative control. **Supplementary Table 2:** Spot‐blot test results for two paleosera extracted from a dental pulp sample and a dental calculus sample taken from one individual US1326 exhumed from the Charleville‐Mézières site, France, in the 19^th^ century (1810‐1813), reacting with anti‐human antibodies compared with *Staphylococcus aureus* as a positive control and with skimmed milk as a negative control. **Supplementary Table 3:** Spot‐blot test results for two paleosera extracted from a dental pulp sample and a dental calculus sample taken from one individual US1339 exhumed from the Charleville‐Mézières site, France, in the 19^th^ century (1810‐1813), reacting with anti‐human antibodies compared with *Staphylococcus aureus* as a positive control and with skimmed milk as a negative control. **Supplementary Table 4:** Spot‐blot test results for two paleosera extracted from a dental pulp sample and a dental calculus sample taken from one individual US1131 exhumed from the Charleville‐Mézières site, France, in the 19^th^ century (1810‐1813), reacting with anti‐human antibodies compared with *Staphylococcus aureus* as a positive control and with skimmed milk as a negative control. **Supplementary Table 5:** Spot‐blot test results for two paleosera extracted from a dental pulp sample and a dental calculus sample taken from one individual US1221 exhumed from the Charleville‐Mézières site, France, in the 19^th^ century (1810‐1813), reacting with anti‐human antibodies compared with *Staphylococcus aureus* as a positive control and with skimmed milk as a negative control. **Supplementary Table 6:** Spot‐blot test results for two paleosera extracted from a dental pulp sample and a dental calculus sample taken from one individual US1255 exhumed from the Charleville‐Mézières site, France, in the 19^th^ century (1810‐1813), reacting with anti‐human antibodies compared with *Staphylococcus aureus* as a positive control and with skimmed milk as a negative control. **Supplementary Table 7:** Spot‐blot test results for two paleosera extracted from a dental pulp sample and a dental calculus sample taken from one individual US1257 exhumed from the Charleville‐Mézières site, France, in the 19^th^ century (1810‐1813), reacting with anti‐human antibodies compared with *Staphylococcus aureus* as a positive control and with skimmed milk as a negative control. **Supplementary Table 8:** Spot‐blot test results for two paleosera extracted from a dental pulp sample and a dental calculus sample taken from one individual US1287 exhumed from the Charleville‐Mézières site, France, in the 19^th^ century (1810‐1813), reacting with anti‐human antibodies compared with *Staphylococcus aureus* as a positive control and with skimmed milk as a negative control. **Supplementary Table 9:** Spot‐blot test results for two paleosera extracted from a dental pulp sample and a dental calculus sample taken from one individual US1297 exhumed from the Charleville‐Mézières site, France, in the 19^th^ century (1810‐1813), reacting with anti‐human antibodies compared with *Staphylococcus aureus* as a positive control and with skimmed milk as a negative control. **Supplementary Table 10:** Spot‐blot test results for two paleosera extracted from a dental pulp sample and a dental calculus sample taken from one individual US1335 exhumed from the Charleville‐Mézières site, France, in the 19^th^ century (1810‐1813), reacting with anti‐human antibodies compared with *Staphylococcus aureus* as a positive control and with skimmed milk as a negative control.

## Data Availability

The data that support the findings of this study are available in the supporting material of this article.
