# Supplementary material for: IgE Anti‐Beta Coronaviruses Serology in Napoleon Soldiers, France
Source: J Med Virol. 2026 Jan 13;98(1):e70800. doi: 10.1002/jmv.70800 (PMC12797278; doi:10.1002/jmv.70800)
Supplement: Supplementary file 1 — Supplementary Table 1: Spot‐blot test results for two paleosera extracted from a dental pulp sample and a dental calculus sample taken from one individual US1300 exhumed from the Charleville‐Mézières site, France, in the 19th century (1810‐1813), reacting with anti‐ human antibodies compared with Staphylococcus aureus as a positive control and with skimmed milk as a negative control. Supplementary Table 2: Spot‐blot test results for two paleosera extracted from a dental pulp sample and a dental calculus sample taken from one individual US1326 exhumed from the Charleville‐Mézières site, France, in the 19th century (1810‐1813), reacting with anti‐human antibodies compared with Staphylococcus aureus as a positive control and with skimmed milk as a negative control. Supplementary Table 3: Spot‐blot test results for two paleosera extracted from a dental pulp sample and a dental calculus sample taken from one individual US1339 exhumed from the Charleville‐Mézières site, France, in the 19th century (1810‐1813), reacting with anti‐human antibodies compared with Staphylococcus aureus as a positive control and with skimmed milk as a negative control. Supplementary Table 4: Spot‐blot test results for two paleosera extracted from a dental pulp sample and a dental calculus sample taken from one individual US1131 exhumed from the Charleville‐Mézières site, France, in the 19th century (1810‐1813), reacting with anti‐human antibodies compared with Staphylococcus aureus as a positive control and with skimmed milk as a negative control. Supplementary Table 5: Spot‐blot test results for two paleosera extracted from a dental pulp sample and a dental calculus sample taken from one individual US1221 exhumed from the Charleville‐Mézières site, France, in the 19th century (1810‐1813), reacting with anti‐human antibodies compared with Staphylococcus aureus as a positive control and with skimmed milk as a negative control. Supplementary Table 6: Spot‐blot test results for two paleosera extra [file JMV-98-e70800-s001.docx]

**Supplementary Tables**

**Supplementary Table 1.** Spot-blot test results for two paleosera extracted from a dental pulp sample and a dental calculus sample taken from one individual US1300 exhumed from the Charleville-Mézières site, France, in the 19^th^ century (1810-1813), reacting with anti- human antibodies compared with *Staphylococcus aureus* as a positive control and with skimmed milk as a negative control.

| **Antigens** | **SARS-CoV-2** | **SARS-CoV-2 (Marseille 4)** | | | | | | **SARS-CoV-2(Wuhan)** | | | | | | **OC43** | | | | | | **229E** | | | | | |
| --- | --- | --- | --- | --- | --- | --- | --- | --- | --- | --- | --- | --- | --- | --- | --- | --- | --- | --- | --- | --- | --- | --- | --- | --- | --- |
| **Antibodies**  **Samples** | **IgT** | **IgT** | **IgA** | **IgG** | **IgM** | **IgE** | **IgD** | **IgT** | **IgA** | **IgG** | **IgM** | **IgE** | **IgD** | **IgT** | **IgA** | **IgG** | **IgM** | **IgE** | **IgD** | **IgT** | **IgA** | **IgG** | **IgM** | **IgE** | **IgD** |
| **Paleoserum extracted from dental pulp of US1300** | NA | - | - | - | - | - | - | - | - | - | - | + | - | - | - | - | - | + | - | - | - | - | - | - | - |
| **Paleoserum extracted from dental calculus of US 1300** | + | NA | NA | NA | NA | NA | NA | NA | NA | NA | NA | NA | NA | - | NA | NA | NA | NA | NA | - | NA | NA | NA | NA | NA |
| **Negative control (Skimmed milk)** | - | - | - | - | - | - | - | - | - | - | - | - | - | - | - | - | - | - | - | - | - | - | - | - | - |
| **Positive control (*S. aureus*)** | + | + | + | + | + | + | + | + | + | + | + | + | + | + | + | + | + | + | + | + | + | + | + | + | + |

**IgT: IgA+IgG+IgM**

**Supplementary Table 2.** Spot-blot test results for two paleosera extracted from a dental pulp sample and a dental calculus sample taken from one individual US1326 exhumed from the Charleville-Mézières site, France, in the 19^th^ century (1810-1813), reacting with anti-human antibodies compared with *Staphylococcus aureus* as a positive control and with skimmed milk as a negative control.

| **Antigens** | **SARS-CoV-2** | **SARS-CoV-2 (Marseille 4)** | | | | | | **SARS-CoV-2(Wuhan)** | | | | | | **OC43** | | | | | | **229E** | | | | | |
| --- | --- | --- | --- | --- | --- | --- | --- | --- | --- | --- | --- | --- | --- | --- | --- | --- | --- | --- | --- | --- | --- | --- | --- | --- | --- |
| **Antibodies**  **Samples** | **IgT** | **IgT** | **IgA** | **IgG** | **IgM** | **IgE** | **IgD** | **IgT** | **IgA** | **IgG** | **IgM** | **IgE** | **IgD** | **IgT** | **IgA** | **IgG** | **IgM** | **IgE** | **IgD** | **IgT** | **IgA** | **IgG** | **IgM** | **IgE** | **IgD** |
| **Paleoserum extracted from Dental pulp of US1326** | NA | + | - | - | - | - | - | + | - | + | - | + | - | - | - | - | - | + | - | - | - | + | - | - | - |
| **Paleoserum extracted from Dental calculus of US 1326** | + | NA | NA | NA | NA | NA | NA | NA | NA | NA | NA | NA | NA | - | NA | NA | NA | NA | NA | - | NA | NA | NA | NA | NA |
| **Negative control (Skimmed milk)** | - | - | - | - | - | - | - | - | - | - | - | - | - | - | - | - | - | - | - | - | - | - | - | - | - |
| **Positive control (*S. aureus*)** | + | + | + | + | + | + | + | + | + | + | + | + | + | + | + | + | + | + | + | + | + | + | + | + | + |

**IgT: IgA+IgG+IgM**

**Supplementary Table 3.** Spot-blot test results for two paleosera extracted from a dental pulp sample and a dental calculus sample taken from one individual US1339 exhumed from the Charleville-Mézières site, France, in the 19^th^ century (1810-1813), reacting with anti-human antibodies compared with *Staphylococcus aureus* as a positive control and with skimmed milk as a negative control.

| **Antigens** | **SARS-CoV-2** | **SARS-CoV-2 (Marseille 4)** | | | | | | **SARS-CoV-2(Wuhan)** | | | | | | | **OC43** | | | | | | | **229E** | | | | | | |
| --- | --- | --- | --- | --- | --- | --- | --- | --- | --- | --- | --- | --- | --- | --- | --- | --- | --- | --- | --- | --- | --- | --- | --- | --- | --- | --- | --- | --- |
| **Antibodies**  **Samples** | **IgT** | **IgT** | **IgA** | **IgG** | **IgM** | **IgE** | **IgD** | **IgT** | **IgA** | **IgG** | **IgM** | **IgE** | **IgD** | **IgT** | | **IgA** | **IgG** | **IgM** | **IgE** | **IgD** | **IgT** | | **IgA** | **IgG** | **IgM** | **IgE** | **IgD** |  |
| **Paleoserum extracted from Dental pulp of US1339** | NA | - | - | + | - | + | - | + | - | - | - | + | - | + | | - | - | - | + | - | - | | + | - | - | - | - |  |
| **Paleoserum extracted from Dental calculus of US 1339** | - | NA | NA | NA | NA | NA | NA | NA | NA | NA | NA | NA | NA | + | | NA | NA | NA | NA | NA | - | | NA | NA | NA | NA | NA |  |
| **Negative control (Skimmed milk)** | - | - | - | - | - | - | - | - | - | - | - | - | - | - | | - | - | - | - | - | - | | - | - | - | - | - |  |
| **Positive control (*S. aureus*)** | + | + | + | + | + | + | + | + | + | + | + | + | + | + | | + | + | + | + | + | + | | + | + | + | + | + |  |

**IgT: IgA+IgG+IgM**

**Supplementary Table 4.** Spot-blot test results for two paleosera extracted from a dental pulp sample and a dental calculus sample taken from one individual US1131 exhumed from the Charleville-Mézières site, France, in the 19^th^ century (1810-1813), reacting with anti-human antibodies compared with *Staphylococcus aureus* as a positive control and with skimmed milk as a negative control.

| **Antigens** | **SARS-CoV-2** | **SARS-CoV-2 (Marseille 4)** | | | | | | **SARS-CoV-2(Wuhan)** | | | | | | | **OC43** | | | | | | | **229E** | | | | | | |
| --- | --- | --- | --- | --- | --- | --- | --- | --- | --- | --- | --- | --- | --- | --- | --- | --- | --- | --- | --- | --- | --- | --- | --- | --- | --- | --- | --- | --- |
| **Antibodies**  **Samples** | **IgT** | **IgT** | **IgA** | **IgG** | **IgM** | **IgE** | **IgD** | **IgT** | **IgA** | **IgG** | **IgM** | **IgE** | **IgD** | **IgT** | | **IgA** | **IgG** | **IgM** | **IgE** | **IgD** | **IgT** | | **IgA** | **IgG** | **IgM** | **IgE** | **IgD** |  |
| **Paleoserum extracted from Dental pulp of US1131** | NA | - | - | - | - | - | - | - | - | - | - | - | - | - | | - | - | - | - | - | - | | - | - | - | - | - |  |
| **Paleoserum extracted from Dental calculus of US 1131** | - | NA | NA | NA | NA | NA | NA | NA | NA | NA | NA | NA | NA | - | | NA | NA | NA | NA | NA | - | | NA | NA | NA | NA | NA |  |
| **Negative control (Skimmed milk)** | - | - | - | - | - | - | - | - | - | - | - | - | - | - | | - | - | - | - | - | - | | - | - | - | - | - |  |
| **Positive control (*S. aureus*)** | + | + | + | + | + | + | + | + | + | + | + | + | + | + | | + | + | + | + | + | + | | + | + | + | + | + |  |

**IgT: IgA+IgG+IgM**

**Supplementary Table 5.** Spot-blot test results for two paleosera extracted from a dental pulp sample and a dental calculus sample taken from one individual US1221 exhumed from the Charleville-Mézières site, France, in the 19^th^ century (1810-1813), reacting with anti-human antibodies compared with *Staphylococcus aureus* as a positive control and with skimmed milk as a negative control.

| **Antigens** | **SARS-CoV-2** | **SARS-CoV-2 (Marseille 4)** | | | | | | **SARS-CoV-2(Wuhan)** | | | | | | | **OC43** | | | | | | | **229E** | | | | | | |
| --- | --- | --- | --- | --- | --- | --- | --- | --- | --- | --- | --- | --- | --- | --- | --- | --- | --- | --- | --- | --- | --- | --- | --- | --- | --- | --- | --- | --- |
| **Antibodies**  **Samples** | **IgT** | **IgT** | **IgA** | **IgG** | **IgM** | **IgE** | **IgD** | **IgT** | **IgA** | **IgG** | **IgM** | **IgE** | **IgD** | **IgT** | | **IgA** | **IgG** | **IgM** | **IgE** | **IgD** | **IgT** | | **IgA** | **IgG** | **IgM** | **IgE** | **IgD** |  |
| **Paleoserum extracted from Dental pulp of US1221** | NA | - | - | - | - | - | - | - | - | - | - | - | - | - | | - | - | - | - | - | - | | - | - | - | - | - |  |
| **Paleoserum extracted from Dental calculus of US 1221** | - | NA | NA | NA | NA | NA | NA | NA | NA | NA | NA | NA | NA | - | | NA | NA | NA | NA | NA | - | | NA | NA | NA | NA | NA |  |
| **Negative control (Skimmed milk)/** | - | - | - | - | - | - | - | - | - | - | - | - | - | - | | - | - | - | - | - | - | | - | - | - | - | - |  |
| **Positive control (*S. aureus*)** | + | + | + | + | + | + | + | + | + | + | + | + | + | + | | + | + | + | + | + | + | | + | + | + | + | + |  |

**IgT: IgA+IgG+IgM**

**Supplementary Table 6.** Spot-blot test results for two paleosera extracted from a dental pulp sample and a dental calculus sample taken from one individual US1255 exhumed from the Charleville-Mézières site, France, in the 19^th^ century (1810-1813), reacting with anti-human antibodies compared with *Staphylococcus aureus* as a positive control and with skimmed milk as a negative control.

| **Antigens** | **SARS-CoV-2** | **SARS-CoV-2 (Marseille 4)** | | | | | | **SARS-CoV-2(Wuhan)** | | | | | | | **OC43** | | | | | | | **229E** | | | | | | |
| --- | --- | --- | --- | --- | --- | --- | --- | --- | --- | --- | --- | --- | --- | --- | --- | --- | --- | --- | --- | --- | --- | --- | --- | --- | --- | --- | --- | --- |
| **Antibodies**  **Samples** | **IgT** | **IgT** | **IgA** | **IgG** | **IgM** | **IgE** | **IgD** | **IgT** | **IgA** | **IgG** | **IgM** | **IgE** | **IgD** | **IgT** | | **IgA** | **IgG** | **IgM** | **IgE** | **IgD** | **IgT** | | **IgA** | **IgG** | **IgM** | **IgE** | **IgD** |  |
| **Paleoserum extracted from Dental pulp of US1255** | NA | - | - | - | - | - | - | - | - | - | - | - | - | - | | - | - | - | - | - | - | | - | - | - | - | - |  |
| **Paleoserum extracted from Dental calculus of US 1255** | - | NA | NA | NA | NA | NA | NA | NA | NA | NA | NA | NA | NA | - | | NA | NA | NA | NA | NA | - | | NA | NA | NA | NA | NA |  |
| **Negative control (Skimmed milk)** | - | - | - | - | - | - | - | - | - | - | - | - | - | - | | - | - | - | - | - | - | | - | - | - | - | - |  |
| **Positive control (*S. aureus*)** | + | + | + | + | + | + | + | + | + | + | + | + | + | + | | + | + | + | + | + | + | | + | + | + | + | + |  |

**IgT: IgA+IgG+IgM**

**Supplementary Table 7.** Spot-blot test results for two paleosera extracted from a dental pulp sample and a dental calculus sample taken from one individual US1257 exhumed from the Charleville-Mézières site, France, in the 19^th^ century (1810-1813), reacting with anti-human antibodies compared with *Staphylococcus aureus* as a positive control and with skimmed milk as a negative control.

| **Antigens** | **SARS-CoV-2** | **SARS-CoV-2 (Marseille 4)** | | | | | | **SARS-CoV-2(Wuhan)** | | | | | | | **OC43** | | | | | | | **229E** | | | | | | |
| --- | --- | --- | --- | --- | --- | --- | --- | --- | --- | --- | --- | --- | --- | --- | --- | --- | --- | --- | --- | --- | --- | --- | --- | --- | --- | --- | --- | --- |
| **Antibodies**  **Samples** | **IgT** | **IgT** | **IgA** | **IgG** | **IgM** | **IgE** | **IgD** | **IgT** | **IgA** | **IgG** | **IgM** | **IgE** | **IgD** | **IgT** | | **IgA** | **IgG** | **IgM** | **IgE** | **IgD** | **IgT** | | **IgA** | **IgG** | **IgM** | **IgE** | **IgD** |  |
| **Paleoserum extracted from Dental pulp of US1257** | NA | - | - | - | - | - | - | - | - | - | - | - | - | - | | - | - | - | - | - | - | | - | - | - | - | - |  |
| **Paleoserum extracted from Dental calculus of US 1257** | - | NA | NA | NA | NA | NA | NA | NA | NA | NA | NA | NA | NA | - | | NA | NA | NA | NA | NA | - | | NA | NA | NA | NA | NA |  |
| **Negative control (Skimmed milk)** | - | - | - | - | - | - | - | - | - | - | - | - | - | - | | - | - | - | - | - | - | | - | - | - | - | - |  |
| **Positive control (*S. aureus*)** | + | + | + | + | + | + | + | + | + | + | + | + | + | + | | + | + | + | + | + | + | | + | + | + | + | + |  |

**IgT: IgA+IgG+IgM**

**Supplementary Table 8.** Spot-blot test results for two paleosera extracted from a dental pulp sample and a dental calculus sample taken from one individual US1287 exhumed from the Charleville-Mézières site, France, in the 19^th^ century (1810-1813), reacting with anti-human antibodies compared with *Staphylococcus aureus* as a positive control and with skimmed milk as a negative control.

| **Antigens** | **SARS-CoV-2** | **SARS-CoV-2 (Marseille 4)** | | | | | | **SARS-CoV-2(Wuhan)** | | | | | | | **OC43** | | | | | | | **229E** | | | | | | |
| --- | --- | --- | --- | --- | --- | --- | --- | --- | --- | --- | --- | --- | --- | --- | --- | --- | --- | --- | --- | --- | --- | --- | --- | --- | --- | --- | --- | --- |
| **Antibodies**  **Samples** | **IgT** | **IgT** | **IgA** | **IgG** | **IgM** | **IgE** | **IgD** | **IgT** | **IgA** | **IgG** | **IgM** | **IgE** | **IgD** | **IgT** | | **IgA** | **IgG** | **IgM** | **IgE** | **IgD** | **IgT** | | **IgA** | **IgG** | **IgM** | **IgE** | **IgD** |  |
| **Paleoserum extracted from Dental pulp of US1287** | NA | - | - | - | - | - | - | - | - | - | - | - | - | - | | - | - | - | - | - | - | | - | - | - | - | - |  |
| **Paleoserum extracted from Dental calculus of US 1287** | - | NA | NA | NA | NA | NA | NA | NA | NA | NA | NA | NA | NA | - | | NA | NA | NA | NA | NA | - | | NA | NA | NA | NA | NA |  |
| **Negative control (Skimmed milk)** | - | - | - | - | - | - | - | - | - | - | - | - | - | - | | - | - | - | - | - | - | | - | - | - | - | - |  |
| **Positive control (*S. aureus*)** | + | + | + | + | + | + | + | + | + | + | + | + | + | + | | + | + | + | + | + | + | | + | + | + | + | + |  |

**IgT: IgA+IgG+IgM**

**Supplementary Table 9.** Spot-blot test results for two paleosera extracted from a dental pulp sample and a dental calculus sample taken from one individual US1297 exhumed from the Charleville-Mézières site, France, in the 19^th^ century (1810-1813), reacting with anti-human antibodies compared with *Staphylococcus aureus* as a positive control and with skimmed milk as a negative control.

| **Antigens** | **SARS-CoV-2** | **SARS-CoV-2 (Marseille 4)** | | | | | | **SARS-CoV-2(Wuhan)** | | | | | | | **OC43** | | | | | | | **229E** | | | | | | |
| --- | --- | --- | --- | --- | --- | --- | --- | --- | --- | --- | --- | --- | --- | --- | --- | --- | --- | --- | --- | --- | --- | --- | --- | --- | --- | --- | --- | --- |
| **Antibodies**  **Samples** | **IgT** | **IgT** | **IgA** | **IgG** | **IgM** | **IgE** | **IgD** | **IgT** | **IgA** | **IgG** | **IgM** | **IgE** | **IgD** | **IgT** | | **IgA** | **IgG** | **IgM** | **IgE** | **IgD** | **IgT** | | **IgA** | **IgG** | **IgM** | **IgE** | **IgD** |  |
| **Paleoserum extracted from Dental pulp of US1297** | NA | - | - | - | - | - | - | - | - | - | - | - | - | - | | - | - | - | - | - | - | | - | - | - | - | - |  |
| **Paleoserum extracted from Dental calculus of US 1297** | - | NA | NA | NA | NA | NA | NA | NA | NA | NA | NA | NA | NA | - | | NA | NA | NA | NA | NA | - | | NA | NA | NA | NA | NA |  |
| **Negative control (Skimmed milk)** | - | - | - | - | - | - | - | - | - | - | - | - | - | - | | - | - | - | - | - | - | | - | - | - | - | - |  |
| **Positive control (*S. aureus*)** | + | + | + | + | + | + | + | + | + | + | + | + | + | + | | + | + | + | + | + | + | | + | + | + | + | + |  |

**IgT: IgA+IgG+IgM**

**Supplementary Table 10.** Spot-blot test results for two paleosera extracted from a dental pulp sample and a dental calculus sample taken from one individual US1335 exhumed from the Charleville-Mézières site, France, in the 19^th^ century (1810-1813), reacting with anti-human antibodies compared with *Staphylococcus aureus* as a positive control and with skimmed milk as a negative control.

| **Antigens** | **SARS-CoV-2** | **SARS-CoV-2 (Marseille 4)** | | | | | | **SARS-CoV-2(Wuhan)** | | | | | | | **OC43** | | | | | | | **229E** | | | | | | |
| --- | --- | --- | --- | --- | --- | --- | --- | --- | --- | --- | --- | --- | --- | --- | --- | --- | --- | --- | --- | --- | --- | --- | --- | --- | --- | --- | --- | --- |
| **Antibodies**  **Samples** | **IgT** | **IgT** | **IgA** | **IgG** | **IgM** | **IgE** | **IgD** | **IgT** | **IgA** | **IgG** | **IgM** | **IgE** | **IgD** | **IgT** | | **IgA** | **IgG** | **IgM** | **IgE** | **IgD** | **IgT** | | **IgA** | **IgG** | **IgM** | **IgE** | **IgD** |  |
| **Paleoserum extracted from Dental pulp of US1335** | NA | - | - | - | - | - | - | - | - | - | - | - | - | - | | - | - | - | - | - | - | | - | - | - | - | - |  |
| **Paleoserum extracted from Dental calculus of US 1335** | - | NA | NA | NA | NA | NA | NA | NA | NA | NA | NA | NA | NA | - | | NA | NA | NA | NA | NA | - | | NA | NA | NA | NA | NA |  |
| **Negative control (Skimmed milk)** | - | - | - | - | - | - | - | - | - | - | - | - | - | - | | - | - | - | - | - | - | | - | - | - | - | - |  |
| **Positive control (*S. aureus*)** | + | + | + | + | + | + | + | + | + | + | + | + | + | + | | + | + | + | + | + | + | | + | + | + | + | + |  |

**IgT: IgA+IgG+IgM**
